# Identification of blood-based key biomarker and immune infiltration in Immunoglobulin A nephropathy by comprehensive bioinformatics analysis and a cohort validation

**DOI:** 10.1186/s12967-022-03330-w

**Published:** 2022-03-29

**Authors:** Jie Xu, Xiahong Shen, Xing Wei, Jie Ding, Jiaojiao Yuan, Zhen Weng, Yang He

**Affiliations:** 1 MOH Key Lab of Thrombosis and Hemostasis, Jiangsu Institute of Hematology, The First Affiliated Hospital of Soochow University, Suzhou, 215006 China; 2 Department of Nephrology, The First Affiliated Hospital of Soochow University, Suzhou, 215006 China; 3 Department of Infectious Disease, Nantong First People’s Hospital, The Second Affiliated Hospital of Nantong University, Jiangsu, 226001 China; 4 Department of Laboratory Medicine, The First Affiliated Hospital of Soochow University, Suzhou, 215006 China; 5 MOE Engineering Center of Hematological Disease, Soochow University, Suzhou, 215123 China; 6 National Clinical Research Center for Hematologic Diseases, The First Affiliated Hospital of Soochow University, Suzhou, 215006 China; 7 Collaborative Innovation Center of Hematology, Soochow University, Suzhou, 215006 China; 8 Cyrus Tang Hematology Center, Soochow University, Suzhou, 215123 China

**Keywords:** Immunoglobulin A, Nephropathy, WGCNA, Immune cell infiltration, Clinical correlated genes

## Abstract

**Background:**

To identify the critical genes in the onset and progression of Immunoglobulin A nephropathy (IgAN) and to explore its immune cell infiltration feature.

**Methods:**

Differentially expressed genes (DEGs) were firstly screened from 1 blood-derived dataset GSE73953 and a glomerulus derived dataset GSE93798 through limma analysis, overlap genes omitting and weighted gene correlation network analysis (WGCNA) and further reduced according to expression pattern and correlation with the clinical features: eGFR and proteinuria, followed by external validation using the GSE37460 dataset and an IgAN cohort. In addition, the CIBERSORT tool for immune cell infiltration analysis, ceRNA network construction and Connectivity Map (CMAP) were also performed.

**Results:**

A total of 195 DEGs were found, and among them, 3 upregulated (ORMDL2, NRP1, and COL4A1) and 3 downregulated genes (ST13, HSPA8 and PKP4) are verified to correlate clinically, and finally ORMDL2, NRP1 and COL4A1 were validated in patient cohort and with the ability of IgAN discrimination (highest AUC was COL4A1: 97.14%). The immune cell infiltration results revealed that significant differences could be found on resting memory CD4 T cells, activated NK cells, and M2 macrophages between control and IgAN.

**Conclusions:**

Our results demonstrated here that significantly upregulated DEGs: ORMDL2, NRP1 and COL4A1, could be served as the diagnostic marker for IgAN, and dysregulated immune cell infiltration hinted possible the immune system intervention point in the setting of IgAN.

**Supplementary Information:**

The online version contains supplementary material available at 10.1186/s12967-022-03330-w.

## Background

Immunoglobulin A nephropathy (IgAN), characterized by glomerular IgA deposits on renal biopsy, is one of the most common primary glomerular disease and remains a leading cause of chronic kidney disease and end-stage kidney disease (ESKD) [1–3]. The common clinical manifestations include hematuria, fever and different degree of proteinuria [4, 5]. The estimated incidence of IgAN is about 2–10 per 100,000 person-years [6, 7] and a higher disease burden has been reported in the East Asian countries, including China [2]. To realize quick diagnosis using a less invasive methodology, blood-based biomarkers rather than renal biopsy that could be more preference in early indication and urgently needed in the clinical practice.

Recent advances in the microarray and transcriptomic technology allow us to obtain a landscape view of the differentially expressed genes (DEGs) in a certain kind of disease [8–10]. Several studies have been performed to assess the expression profiling of the samples from IgAN. For example, Nagasawa et al. [11] performed the microarray using 15 IgAN patients derived peripheral blood mononuclear cells (PBMCs), Liu et al. [12] employed microarray analysis of the glomerular compartment of renal biopsy specimens from 19 IgAN patients and Guo et al. [13] used microarrays to analyze the transcriptome of microdissected renal biopsies from 27 patients with IgAN. However, certain bias could be presented in these isolated experiments due to the complicated human genetic background and different experimental conditions. Integrative bioinformatics analysis can provide the possibility of multiple group verification of the DEGs, thereby facilitating the process of novel and reliable biomarkers identification.

In the present study, we aimed to identify the critical genes in the onset and progression of IgAN via integrative employment of blood-based microarray data and glomerular tissue-related data, thereby identifying the novel biomarkers for IgAN diagnosis. Moreover, we also explored immune cell infiltration feature using the glomerular-related data to discover the possible treatment intervention for IgAN.

## Methods

### Patients recruitment and clinical data collection

A total of 7 IgAN were recruited from the Department of Nephrology, the First Affiliated Hospital of Soochow University for peripheral blood mononuclear cells (PBMCs) collection from June 2021 to September 2021. The diagnosis of IgAN were based on The Oxford Classification for IgAN and our previous publication [14, 15], and all the included IgAN patients were primarily confirmed with histological features of deposition of IgA in the mesangial area by immunofluorescence. Patients who were presented with secondary IgAN, such as allergic purpura, chronic hepatitis B, were excluded. The clinical data include gender, serum creatinine, estimated glomerular filtration rate (eGFR), and 24 h proteinuria was collected (Table 1). Moreover, PBMCs were also obtained from 5 healthy volunteers as controls. The current study protocol was approved by the Ethical Committee of the First Affiliated Hospital of Soochow University and adhered to the requirement of the Declaration of Helsinki. All the subjects were required to provide written informed consent.

**Table 1:** Patients characteristics

Parameters	Healthy controls (n = 5)	IgAN (n = 7)
Age (mean, range)	44.8 (38–54)	39.4 (24–62)
Gender (female, %)	3(60%)	3(43%)
Serum creatinine (mean ± SD, µmol/L)	67.12 ± 15.28	109.77 ± 21.20
eGFR (mean ± SD, mL/min/1.73 m^2^)	103.91 ± 8.09 (94.56–115.23)	65.78 ± 6.77
24 h urine protein (mean ± SD, g/24 h)	Negative^a^	0.86 ± 0.57

*IgAN* IgA nephropathy, *eGFR* estimated glomerular filtration rate

^a^Negative in the qualitative test

### Microarray data and related clinical data acquisition

The mRNA expression and related clinical data about IgAN were downloaded from Gene Expression Omnibus (GEO) (http://www.ncbi.nlm.nih.gov/geo/) using the search terms “Immunoglobulin A nephropathy”, “IgA nephropathy”, “IgAN” and “expression profiling by array”. The gene expression microarray datasets GSE73953, GSE93798 and GSE37460 were selected and downloaded. The criteria for dataset selection were as follows: human clinical samples with detailed clinical and gene expression information. Among these datasets, GSE73953 and GSE93798 were used for differentially expressed genes (DEGs) screening, and patient eGFR and proteinuria data downloaded from The Nephroseq v5 analysis engine (https://v5.nephroseq.org) was used for correlated gene screening. GSE37460 and above cohort containing IgAN and control subjects were used for expression-level validation of the 6 identified eGFR and proteinuria correlated genes at different tissue composition levels. Detailed information on these microarray datasets is listed in Additional file 11: Table S1.

### Identification of the candidate genes via integrated bioinformatic analysis

**Fig. 1:**
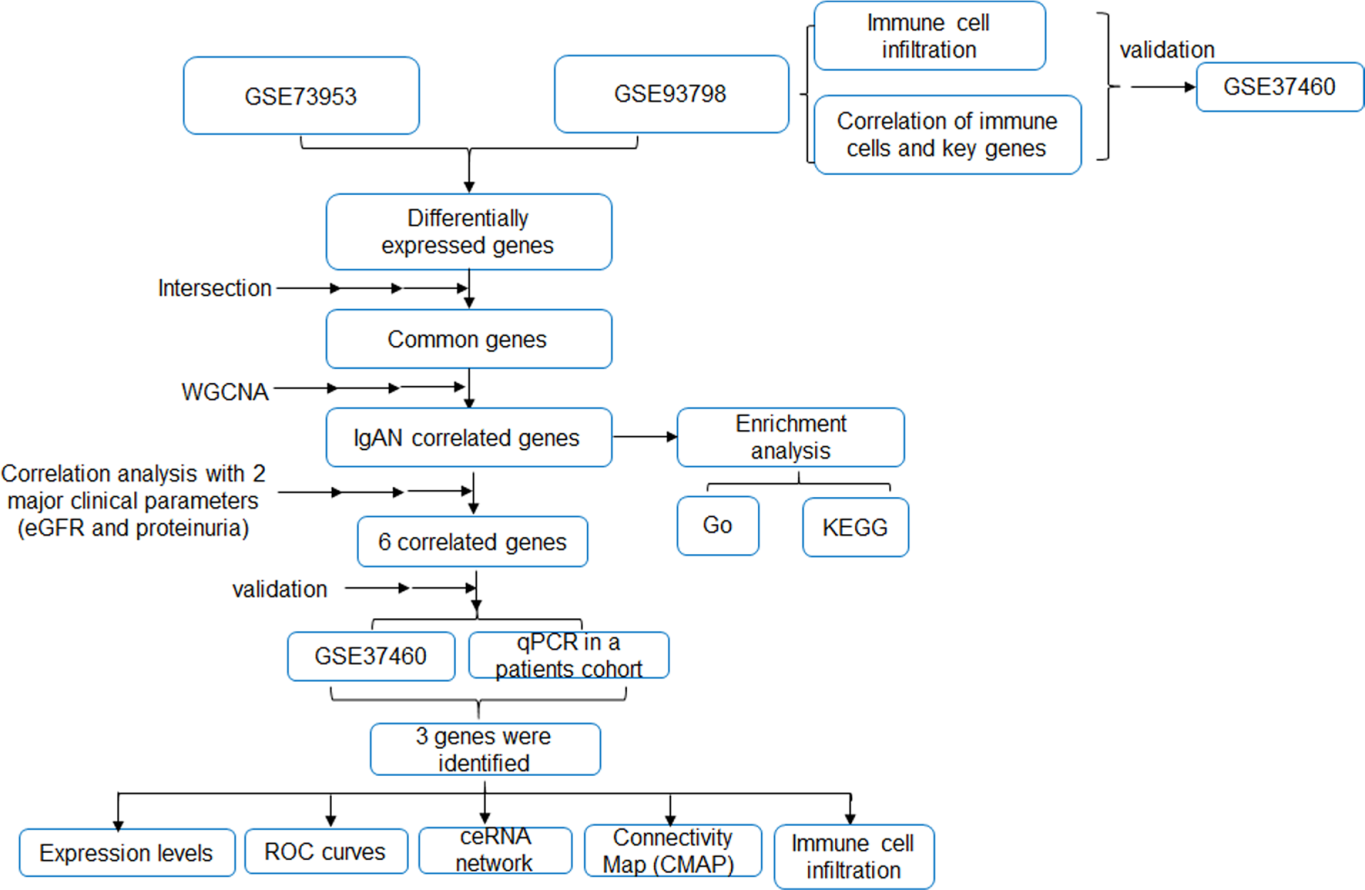
Flow chart to illustrate the analysis process of the present study

The entire process of the analysis was shown in Fig. 1 and the detailed screening of the DEGs contains 3 steps as follows:

#### Data processing

**Fig. 2:**
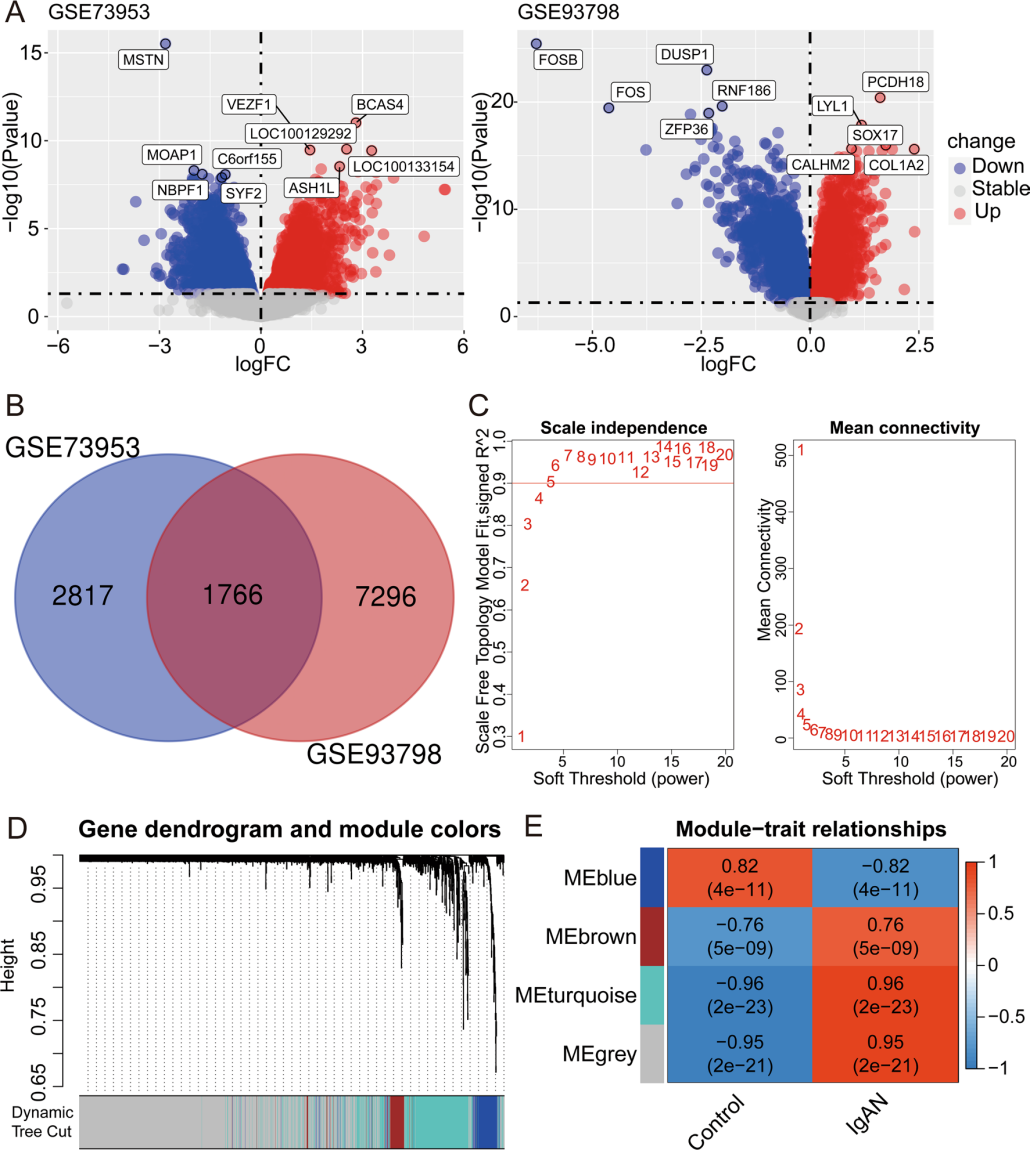
Identification of differentially expressed genes (DEGs) using intersection and weighted gene co-expression network analysis (WGCNA) of the datasets. **A** DEGs were obtained via p-value criteria from GSE73953 (Peripheral blood mononuclear cells) and GSE93798 (Glomerular part of human kidney). **B** Intersection was performed to obtain the common DEGs from **A**. **C** Scale-free topology model to identify the best beta value (β = 5) using the intersected common genes. **D** Gene dendrogram and module colors. **E** Module-trait relationship

The “limma” package in R [16] (http://www.bioconductor.org/) was used for background correction, normalization and differential expression analysis using IgAN and control samples from GSE73953 and GSE93798. Initial screening of the DEG was performed using the p < 0.05 as the criteria, and was illustrated as Venn maps via an online tool (http://bioinformatics.psb.ugent.be/webtools/Venn/) (Fig. 2A). Then the intersected DEGs from the 2 datasets were employed in the following analysis (Fig. 2B).

#### Construction of weighted gene co-expression network analysis (WGCNA)

The “WGCNA” package in R [17] was employed for the biologically meaningful module information mining based on pairwise correlations between genes from high-throughput data. Co-expression networks were built using the intersected DEGs and corresponding clinical information from above step to illustrate strong and weak correlations between genes. To fit for the scale-free network, the square of the between genes correlation coefficient was calculated to pick up the optimal soft threshold 5 (Fig. 2C, left), followed by the mean connectivity calculation of the corresponding soft threshold (Fig. 2C, right). Then, the adjacency and topological overlap matrix (TOM) similarity matrices were generated based on the selected soft threshold for modules detection (generating dendrogram), followed by the module assignment under the dendrogram (Fig. 2D) and correlation of the modules to the clinical traits to identify the important DEGs (Fig. 2E).

#### Clinical correlated genes identification and functional enrichment analysis

The Nephroseq v5 analysis engine (https://v5.nephroseq.org) provides the gene expression features and clinical characteristics. Pearson correlation analysis was performed using the DEGs from 2.3.2 to identify the genes that correlated with eGFR and proteinuria. Finally, protective genes with a definition of eGFR negatively correlated and proteinuria positively correlated were selected.

Gene Ontology (GO) annotation and Kyoto Encyclopedia of Genes and Genomes (KEGG) pathway enrichment analyses were performed with the “clusterProfiler” package in R [18]. Disease Ontology (DO) enrichment analyses were performed on DEGs using the “clusterProfiler” and “DOSE” packages in R [19].

### Immune cells infiltration analysis

To quantify the relative proportions of infiltrating immune cells based on the gene expression matrices from above IgAN datasets (GSE93798 and GSE37460), a bioinformatics algorithm named CIBERSORT (https://cibersortx.stanford.edu/) [20] was employed for immune cell infiltrations calculation. The putative abundance of immune cells was estimated using a reference set with 22 types of immune cell subtypes (LM22) with 1,000 permutations. Violin plots were drawn using the “ggplot2” package in R to visualize the differences in immune cell infiltration between the IgAN and control samples. Correlation analysis and visualization of 22 types of infiltrating immune cells were performed using the “corrplot” package in R. Moreover, the relationships between the clinical correlated DEGs and 22 types of immune cells were also visualized.

### ceRNA network construction and connectivity map (CMAP) analysis

The interaction of DEGs identified from 2.3.3 with the possible miRNA were predicted using TargetScan (http://www.targetscan.org) database, and the further interaction between miRNA and circRNA or lncRNA were constructed using StarBase (http://starbase.sysu.edu.cn) database. In order to simplify the interaction links, CircRNAs or lncRNAs candidates with the weak interaction with miRNAs were removed using the ClipExpNum software from StarBase. Moreover, Connectivity Map (https://clue.io/), an online database that relates disease, genes, and drugs based on similar or opposite gene expression signatures, was used for potential drug prediction. Briefly, the DEGs were compared with the reference data from the database to obtain a correlation score (− 100 to 100), and the small molecule compounds with a mean coefficient less than − 98 were ranked and selected.

### Real-time reverse transcription PCR to verification of the clinical related genes

PBMCs were obtained from the IgAN and healthy controls via density gradient centrifugation using the Ficoll reagents from Solarbio (Wuhan, China). RNA extraction was employed the RNAeasy reagent (Vazyme, Nanjing, China) whereas reverse transcription was performed using the HiScript III 1st Strand cDNA Synthesis Kit (Nanjing, China) according to manufacturer’s instruction, and real-time PCR was carried out using SYBR^®^ Green Master Mix (Vazyme, Nanjing, China) on the ABI StepOne PlusTM real-time PCR system. The Relative mRNA expression was calculated using the 2^−ΔΔCt^ method in a triplicated manner. Primers were as described in Table 2.

**Table 2:** Primer information

Target name		Primer
ORMDL2	F	CAGCATTCCTGTTGTCTGGACC
	R	TGTCAGTAGCCGAGCCTTTCCT
NRP1	F	CTGTGAAGTGGAAGCCCCTAC
	R	TGTGAGCTGGAAGTCATCACC
COL4A1	F	TGTTGACGGCTTACCTGGAGAC
	R	GGTAGACCAACTCCAGGCTCTC
ST13	F	AGAAGTTCAACCTAGGGCACAG
	R	GAGCTCCTGACTGTCGTCTG
PKP4	F	TCTGTTCAGGCAAATGCAGCGG
	R	TCTGTGGTCCAGAAGGTCAACC
GAPDH	F	GTCTCCTCTGACTTCAACAGCG
	R	ACCACCCTGTTGCTGTAGCCAA

### Statistical analysis

Statistical analysis was performed using R version 4.1.0. Quantitative data are expressed as the mean ± standard deviation (SD), and qualitative data are expressed as numbers and percentages. Unpaired t-tests and Mann–Whitney U tests were respectively used for parametric and nonparametric 2-group comparisons of quantitative data. Correlation analysis between the clinical parameters and gene expression level was performed using Pearson correlation. The diagnostic efficacy was calculated by receiver operator characteristic (ROC) curves and area under the curve (AUC) using the “survivalROC” and “ROCR” packages in R. Unless specifically mentioned, p < 0.05 was considered statistically significant.

## Results

### Identification of the candidate genes using GEO datasets and gene enrichment analysis

**Fig. 3:**
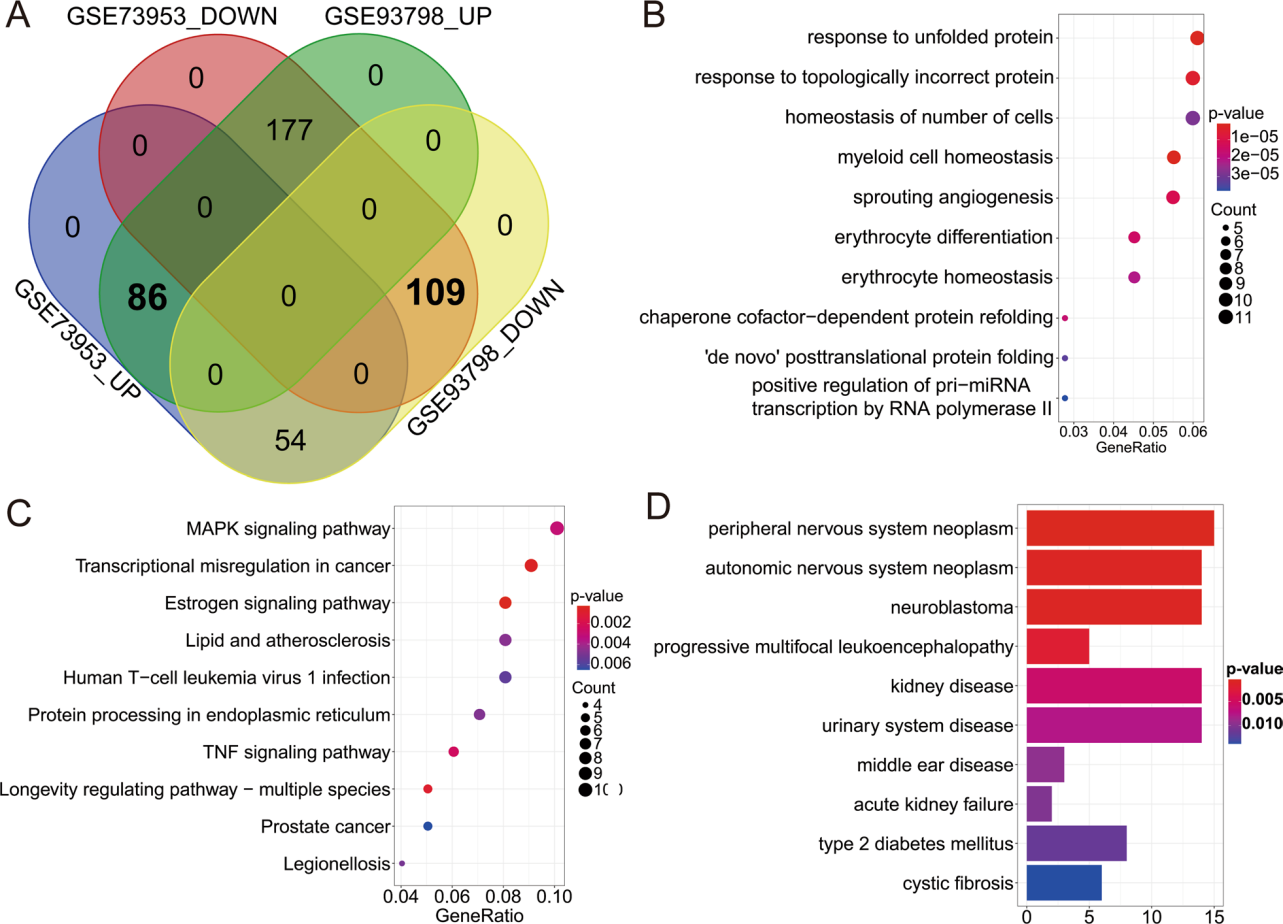
Gene enrichment analysis. **A** The genes from the most relevant modules of above WGCNA were further classified based on the up-or downregulation and intersection was performed to obtain the gene with consistent pattern in 2 datasets (GSE73953 and GSE93798). **B** Top 10 GO term enrichment analysis of the genes from **A**. **C** Top 10 KEGG pathway analysis of the genes from **A**. **D** Disease ontology analysis of the genes from **A**

To find critical genes involved in the IgAN development and progression, we employed the 1 GEO dataset using PBMCs samples (GSE73953) and 1 GEO dataset using the glomerular part of the human kidney (GSE93798) as the training sets to perform the DEGs analysis. After application of the criteria of p < 0.05, a total of 4583 and 9072 genes were respectively found in GSE73953 and GSE93798 (Fig. 2A, B; Additional file 1) and the intersection of the genes are 1766 (Additional file 1). To further decrease the gene number, WGCNA was applied to confirm the IgAN phenotype-related genes (Fig. 2C–E) and a total of 426 genes were found (Additional file 2). After confirmation of the consistent expression pattern in 2 datasets, a total of 86 up- and 109 down-regulated genes were found (Fig. 3A; Additional file 3). Then, we performed the GO enrichment, KEGG pathway and disease ontology analysis (Fig. 3B–D), the results showed that response to unfolded protein, response to topologically incorrect protein and homeostasis of the cell number are the top 3 GO terms, MAPK signaling pathway, transcriptional misregulation in cancer and Estrogen signaling pathway are the top 3 KEGG pathways. For disease ontology terms, peripheral nervous system neoplasm, autonomic nervous system neoplasm and neuroblastoma ranked top 3 terms, and kidney disease and urinary system disease ranked 5 and 6 among the top 10 terms.

### Identification of the genes of clinical significance

**Fig. 4:**
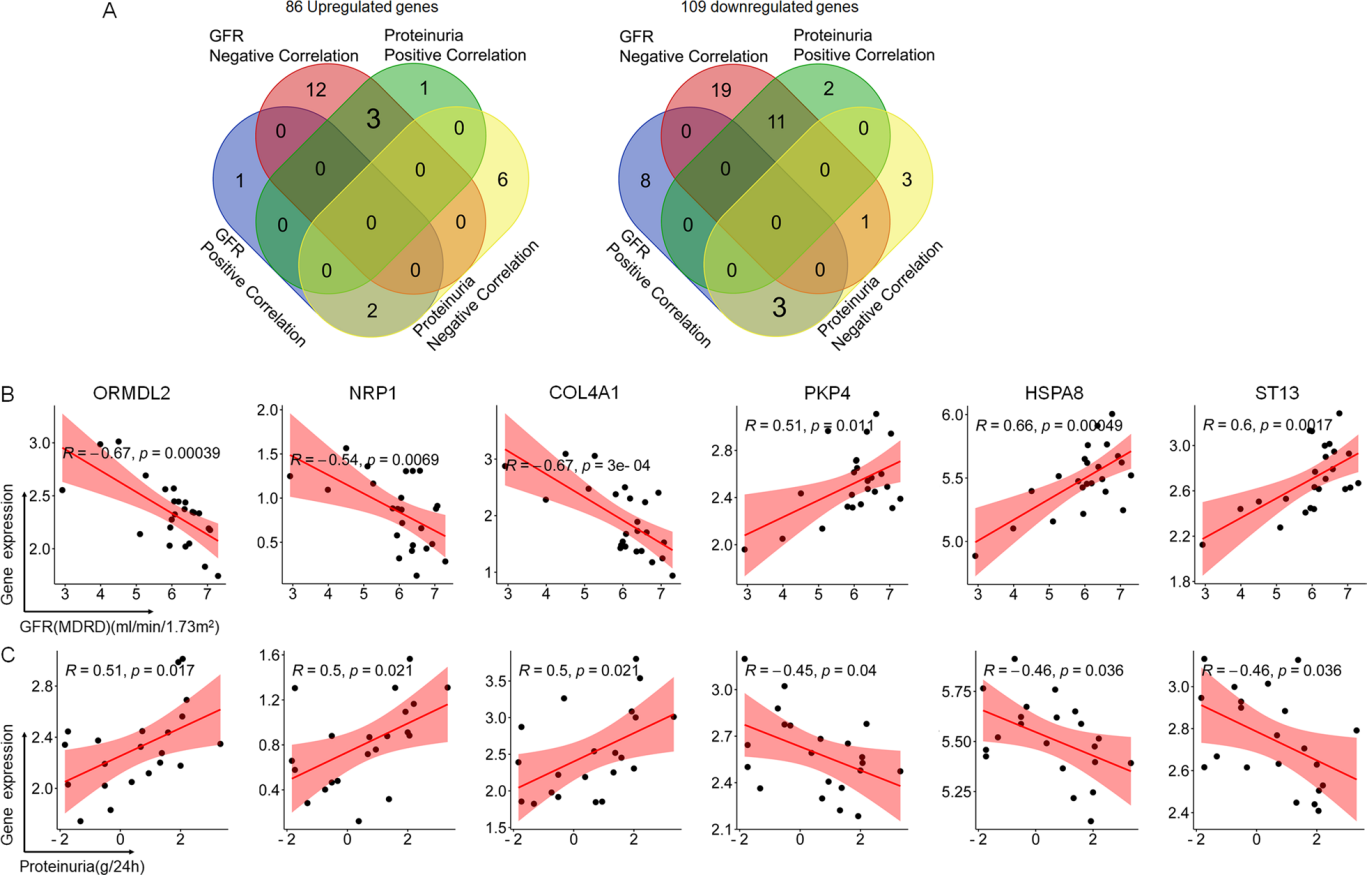
Correlation analysis of DEGs and corresponding clinical characteristics of estimated glomerular filtration rate (eGFR) and proteinuria to identify the protective genes. Protective gene was defined as eGFR positive correlated and proteinuria negative correlated in downregulated genes whereas the opposite trend in upregulated genes. **A** A total of 6 genes were found, including 3 upregulated (left) and 3 downregulated protective genes (right). **B** Correlation maps of above-identified 6 genes were shown, including ORMDL2, NRP1, COL4A1, ST13, HSPA8 and PKP4

Since the Nephroseq v5 analysis engine allows to explore the relationship between gene expression levels and clinical characteristics, we further performed the correlation analysis using the gene expression characteristics and 2 most critical clinical characteristics in IgAN, eGFR and proteinuria (Additional files 4, 5). Using the protective genes defined in the methods part, we totally found 3 upregulated (ORMDL2, NRP1 and COL4A1; Fig. 4A, left) and 3 downregulated protective genes (ST13, HSPA8 and PKP4; Fig. 4A, right). Moreover, we also provided the correlation maps of these 6 genes, and a relatively high correlation parameter R-value (≥ 0.45) and significant p-value (all < 0.05) were found (Fig. 4B). Via above 3-step of integrated bioinformatics analysis and combination of the clinical characteristics, a total of 6 genes were found.

### Validation of the expression pattern and evaluation of diagnostic efficacy of above identified genes

**Fig. 5:**
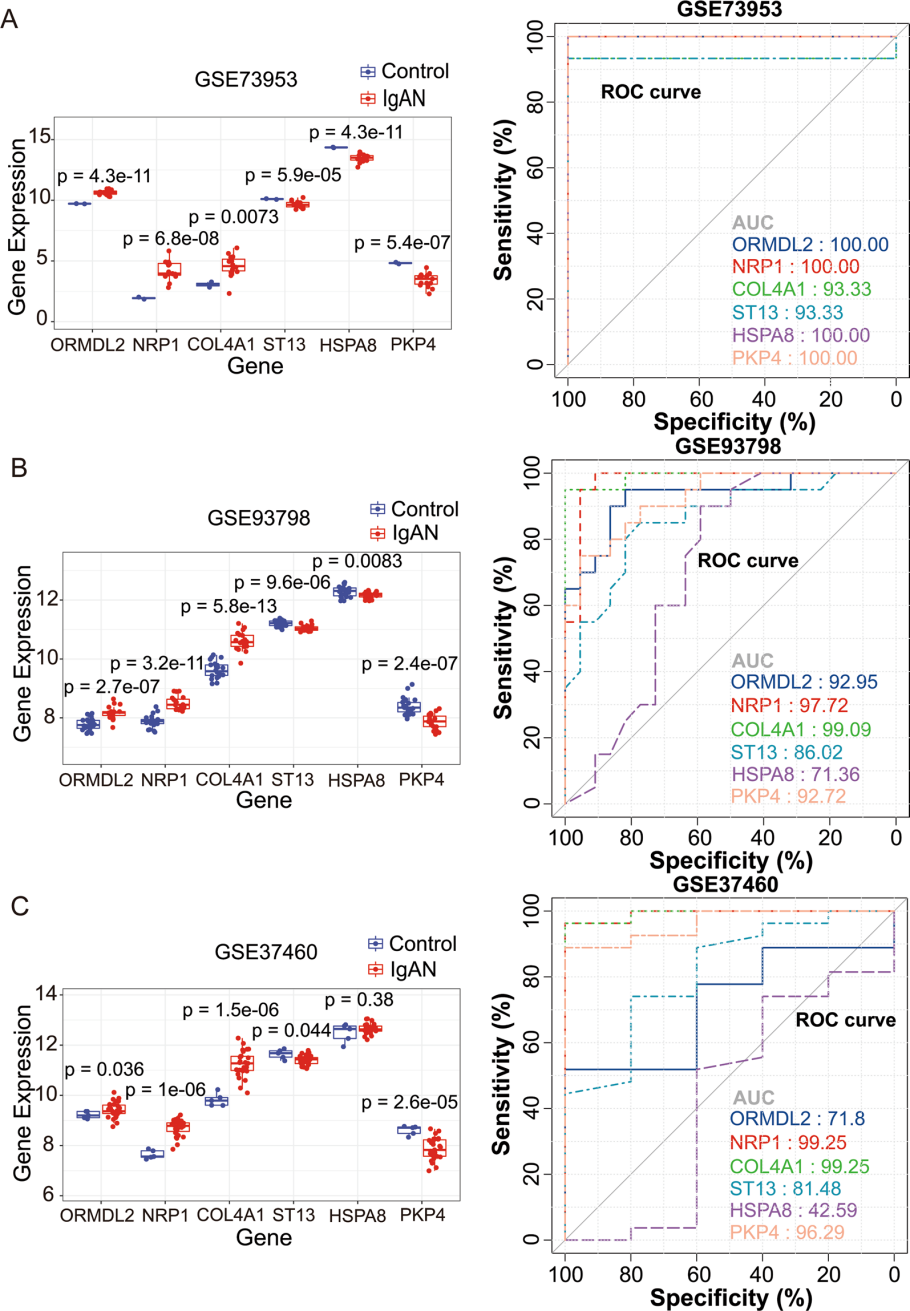
Validation of the expression pattern and evaluation of diagnostic efficacy of 6 clinical correlated genes using the GEO datasets. **A** In the dataset of GSE73953, significantly upregulated genes are ORMDL2, NRP1 and COL4A1, and significantly downregulated genes are ST13, HSPA8 and PKP4. According to the ROC curves, the highest area under curve (AUC) could be observed using the gene ORMDL2, NRP1, HSPA8 and PKP4. **B** In the dataset of GSE93798, the same results were found on significantly upregulated and downregulated genes as that in GSE73953. According to the ROC curves, the highest AUC could be observed using the gene COL4A1. **C** In the dataset of GSE37460, significantly upregulated genes are ORMDL2, NRP1 and COL4A1, and significantly downregulated genes are ST13 and PKP4. According to the ROC curves, the highest AUC could be observed using the gene NRP1 and COL4A1

To further validate the clinical significance of 6 genes, we employed 1 GEO dataset (GEO37460) and a small cohort of patients from our hospital to verify. We firstly confirmed our results in 2 training sets, GSE73953 and GSE93798 (Fig. 5A, B), and the results showed that consistent up- and downregulated expression pattern and high AUC values of all the 6 genes according to the ROC curves. Further validation using the dataset of GSE37460 revealed that significantly upregulated genes were ORMDL2, NRP1 and COL4A1, and significantly downregulated genes were ST13 and PKP4. Therefore, HSPA8 is omitted in the following cohort analysis due to the absence of differences. Moreover, according to the results from ROC curves, highest AUC could be observed using the gene NRP1 and COL4A1 (both are 99.25%; Fig. 5C).

**Fig. 6:**
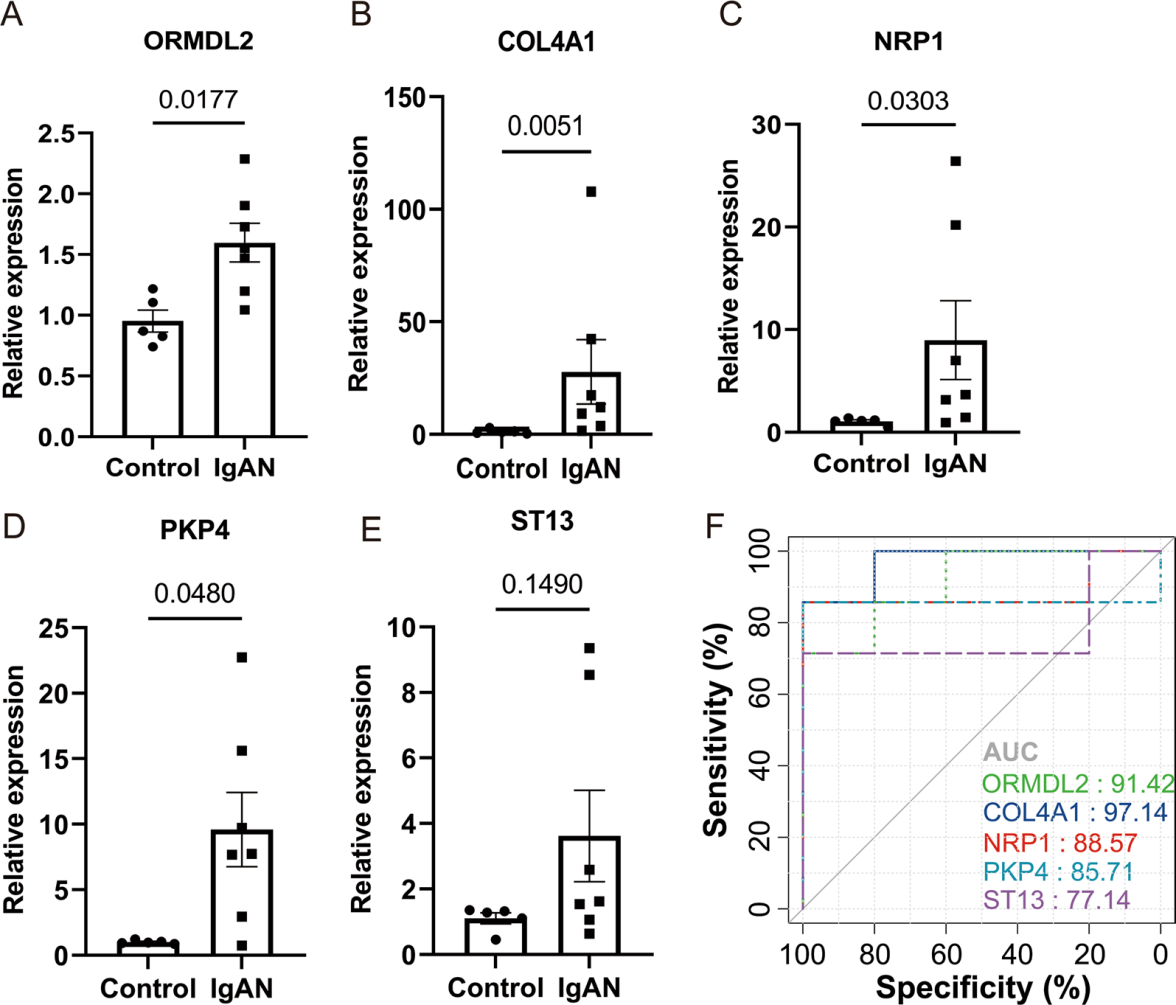
qPCR Validation of the expression pattern and evaluation of diagnostic efficacy of 6 clinical correlated genes using our patient cohort. In our cohort, significantly upregulated genes were confirmed on ORMDL2 (**A**), COL4A1 (**B**) and NRP1 (**C**), whereas PKP4 (**D**) and ST13 (**E**) were not consistent with the results from datasets. According to the ROC curves (**F**), the highest AUC could be observed using the gene COL4A1

In our cohort, DEGs with consistent pattern were only confirmed on 3 upregulated genes, ORMDL2, NRP1 and COL4A1 (Fig. 6A–C), but not on PKP4 and ST13 (Fig. 6D–E). Moreover, according to the ROC curves, highest AUC could be observed using the gene COL4A1 (97.14%) (Fig. 6F). These results suggested that significantly upregulated genes ORMDL2, NRP1 and COL4A1 could be served as the gene marker to differentiate IgAN and controls.

### ceRNA network construction and CMAP results

**Fig. 7:**
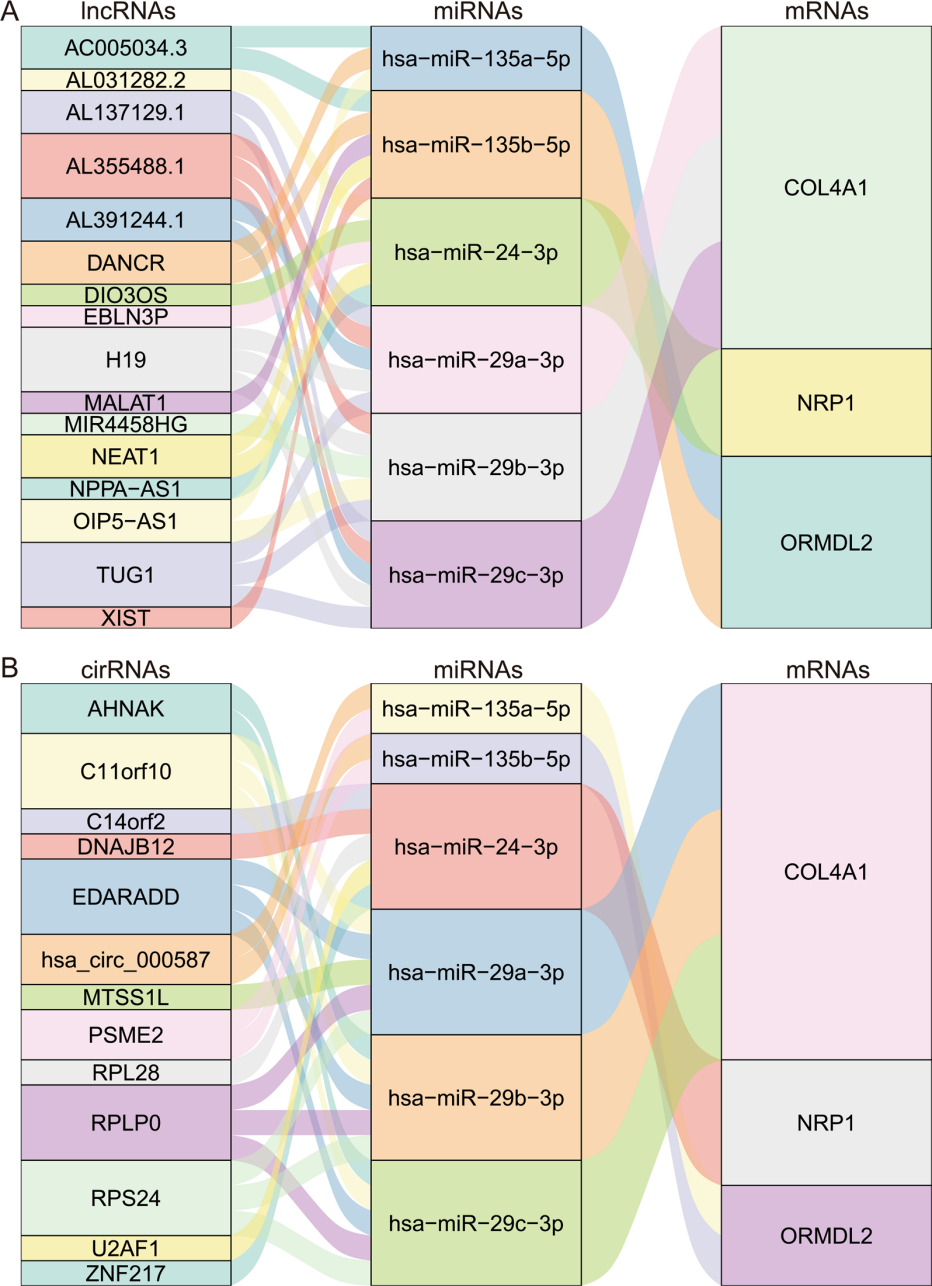
CeRNA network of the 3 key genes were shown using Sankey diagram. A.LncRNAs-miRNAs-mRNAs network, B. CirRNAs-miRNAs-mRNAs network. The connection degree of each gene is visualized according to the size of the rectangle

Since non-coding RNAs (including circRNAs, lncRNA and miRNA) are considered to play an important role in the regulation of the function of mRNA. To better understand the regulatory network of mRNAs found in the above analysis, we constructed 2 ceRNA networks. The results about lncRNA-miRNA-mRNA network and mRNA-miRNA-circ-RNA network are shown in Fig. 7A, B (Additional file 6). Strong interactions could be found in the genes COL4A1, NRP1 and ORMDL2 with 6 miRNAs, 16 lncRNAs and 13 cirRNAs. These data provided a preliminary view of the regulatory network of our identified mRNAs.

Moreover, according to the expression profiling the DEGs from 2.3.3, a total of 17 small-molecule compounds were identified as potential therapeutic agents from CMAP analysis (Table 3; Additional file 7).

**Table 3:** Small molecular compounds identified by connectivity map

Cell ID molecule	A375	A549	HCC515	HEPG2	HT29	MCF7	PC3	HA1E	VCAP	Summary
BNTX	− 99.82	26.26	− 99.4	NaN	− 99.76	− 98.98	− 93.17	− 99.91	− 99.58	− 99.3
MLN-2238	− 99.74	− 99.77	− 97.45	− 99.93	− 99.24	− 97.23	− 99.51	− 88.12	− 98.67	− 99.08
NVP-AUY922	− 97.55	− 99.63	− 80.98	− 99.33	− 92.44	− 99.77	− 98.95	− 99.31	− 99.37	− 99.01
NSC-632839	− 99.81	− 92.34	− 99.56	− 99.88	− 99.09	− 53.98	− 97.23	− 99.61	− 99.5	− 98.98
Calmidazolium	NaN	− 99.55	− 95.85	0	− 99.39	− 99.04	− 99.57	NaN	− 80.09	− 98.91
Homoharringtonine	− 99.47	− 99.52	− 99.68	− 97.24	− 99.5	− 98.45	− 94.95	− 99.67	NaN	− 98.86
QL-XII-47	− 99.74	− 93.89	− 99.5	− 99.88	− 99.38	− 88.94	0	0	0	− 98.8
Geldanamycin	− 95.31	− 99.17	− 96.98	− 97.4	− 95.78	− 99.72	− 99.76	− 99.39	− 99.45	− 98.77
Diphencyprone	− 98.84	− 67.82	NaN	NaN	0	4.47	− 99.21	− 98.88	− 61.33	− 98.7
Puromycin	− 99.18	− 99.65	− 99.06	− 99.56	− 98.09	− 99.71	− 98.41	− 99.74	− 98.29	− 98.66
PU-H71	− 27.33	− 94.9	− 96.15	− 93.12	− 99.15	− 94.61	− 98.88	− 81.98	− 99.37	− 98.48
Verrucarin-a	− 99.11	− 99.41	− 99.53	− 96.35	− 99.03	− 13.53	− 88.33	− 98.88	− 59.74	− 98.31
MG-132	− 98.94	− 99.14	− 99.13	− 99.42	− 98.34	− 97.43	− 99.69	− 99.45	− 92.85	− 98.31
EI-346-erlotinib-analog	− 99.72	− 93.78	− 90.77	− 98.86	39.52	− 98.16	− 65.15	− 99.92	− 80.7	− 98.24
kinetin-riboside	− 98.73	− 98.78	− 97.87	− 99.12	− 94.46	− 95.06	− 92.65	− 99.45	− 54.55	− 98.18
Triciribine	− 91.35	45.62	− 77.55	− 98.88	− 75.13	− 97.27	− 93.95	− 98.95	− 67.77	− 98.17
Narciclasine	− 99.3	− 98.58	− 99.07	0	− 99	− 98.96	− 96.81	− 99.51	0	− 98.06

### Results of immune cell infiltration

**Fig. 8:**
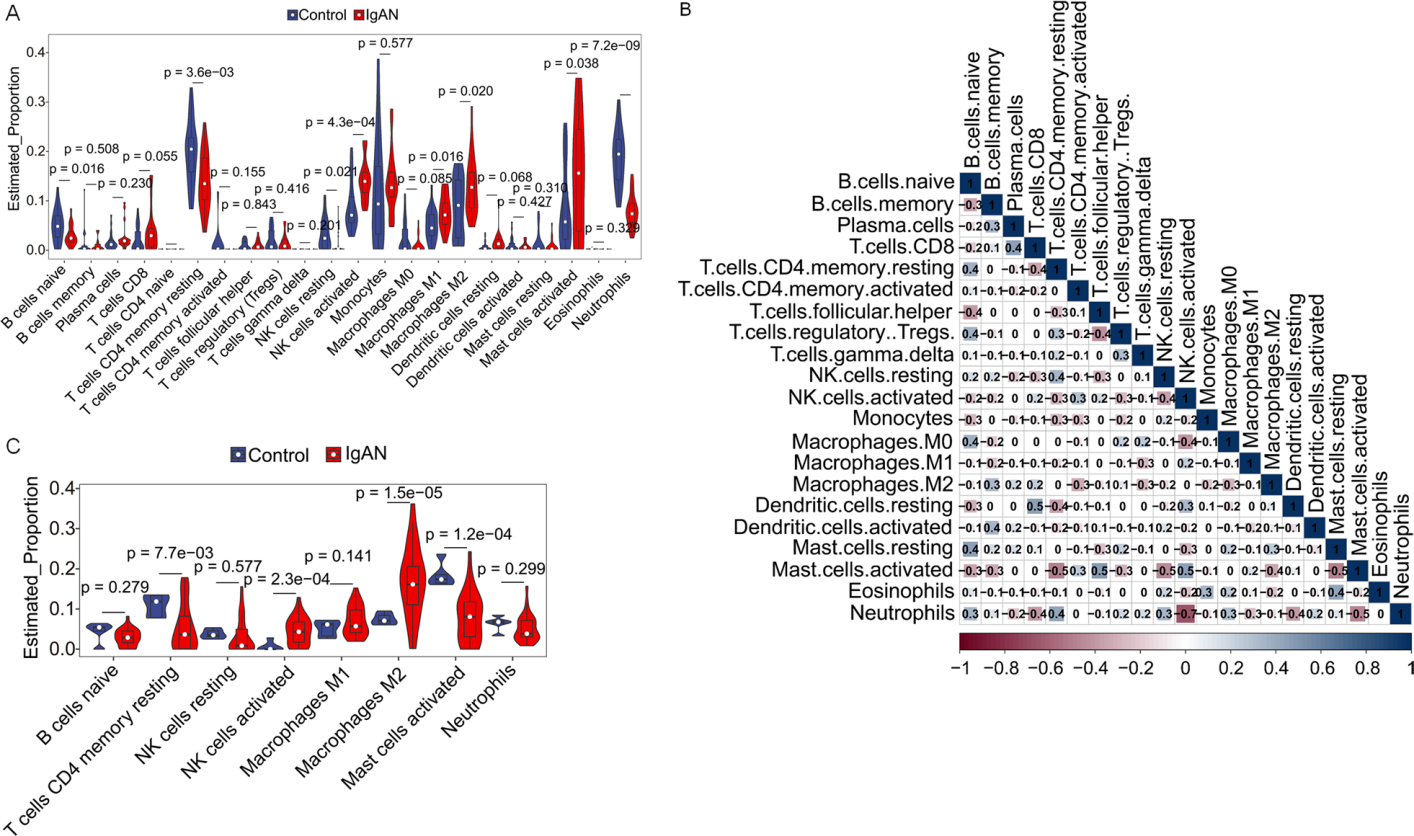
Immune infiltration analysis. **A** Violin plot of the estimated proportion of 22 types of immune cells between control and IgA nephropathy using the dataset of GSE93798. Significant difference could be found on naive B cells, resting memory CD4 T cells, resting and activated NK cells, M1 and M2 macrophages, activated mast cells, and neutrophils. **B** Correlation heat map of 22 types of immune cells. Positive and negative correlation was respectively shown in blue and red color, whereas the number represent the correlation parameters. C. Validation of the immune cell infiltration results using the datasets of GSE37460. Compared with the normal control, resting memory CD4 T cells, activated NK cells, and M2 macrophages were confirmed as difference

The autoimmune disease property of IgAN made it necessary to explore the possible dysregulation of immune cells, whereas the dataset GSE93798 providing the expression profiling data from glomerulus made it possible to fulfill this goal. According to the immune cell infiltration results (Fig. 8A; Additional file 8), significant difference could be found on naive B cells (p = 0.016), resting memory CD4 T cells (p < 0.001), resting and activated NK cells (p = 0.021 and < 0.001), M1 and M2 macrophages (p = 0.016 and 0.020), activated mast cells (p = 0.038), and neutrophils (p < 0.001) between control and IgAN.

Moreover, the correlation of 22 types of immune cells were also calculated, the results revealed that naive B cells were significantly positively correlated with resting memory CD4 T cells (r = 0.37, p = 0.015), regulatory T cells (r = 0.38, p = 0.014), M0 macrophages (r = 0.37, p = 0.016), resting mast cells (r = 0.44, p = 0.003) and neutrophils (r = 0.35, p = 0.021), and negatively correlated with memory B cells (r = − 0.34, p = 0.027), follicular helper T cells (r = -0.42, p = 0.006), resting dendritic cells (r = − 0.34, p = 0.029) and activated mast cells (r = − 0.33, p = 0.033); resting memory CD4 T cells were significantly positively correlated with resting NK cells (r = 0.37, p = 0.017) and neutrophils (r = 0.44, p = 0.003) and negatively correlated with resting dendritic cells (r = − 0.38, p = 0.013) and activated mast cells (r = − 0.49, p < 0.001); resting NK cells were significantly positively correlated with eosinophils (r = 0.39, p = 0.011) and neutrophils (r = 0.37, p = 0.015) and negatively correlated with activated NK cells (r = − 0.41, p = 0.007) and activated mast cells (r = − 0.49, p = 0.001); activated NK cells were significantly positively correlated with resting dendritic cells (r = 0.36, p = 0.018) and activated mast cells (r = 0.45, p = 0.003) and negatively correlated with M0 macrophages (r = − 0.41, p = 0.007) and neutrophils (r = − 0.68, p < 0.001); M2 macrophages were significantly negatively correlated with activated mast cells (r = − 0.35, p = 0.023); and activated mast cells were significantly negatively correlated with neutrophils (r = − 0.46, p = 0.002) (Fig. 8B; Additional file 9).

In addition, we used the external dataset GSE37460 to validate the above identified 8 different infiltrated immune cells between IgAN and normal controls. The violin plot of the immune cell infiltration showed that, compared with the normal control, resting memory CD4 T cells (p < 0.001), activated NK cells (p < 0.001), and M2 macrophages (p < 0.001) were confirmed as difference (Fig. 8C).

### Immune cell infiltration correlation analysis of 3 validated genes

**Fig. 9:**
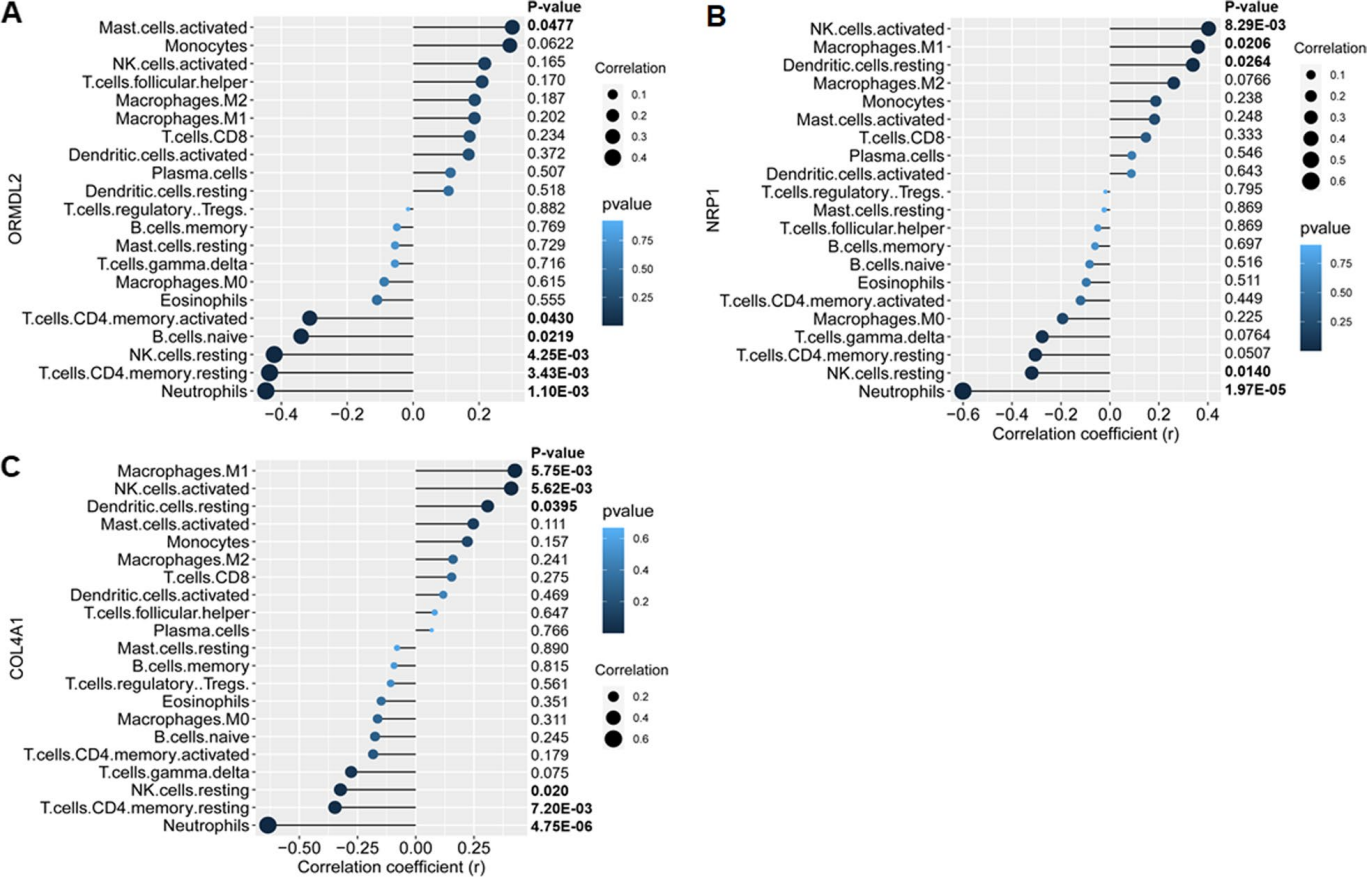
Correlation map of 22 types of immune cells and 3 key genes. A positive and negative correlation was respectively shown in right and left direction, whereas the high and low p-value was respectively shown in light and deep blue color. The size of the circle represents the strength of correlation, the larger of the size, the stronger of the correlation. **A** ORMDL2. **B** NRP1. **C** COL4A1

Furthermore, we also explored the correlations between 3 validated genes and different immune cell types, the results showed that ORMDL2 had positive correlation with activated mast cells (r = 0.31, p = 0.0477), and negative correlations with CD4 memory activated T cells (r =  − 0.31, p = 0.0430), and naive B cells (r =  − 0.35, p = 0.0219), resting NK cells (r =  − 0.44, p < 0.0001), CD4 memory resting T cells (r =  − 0.44, p < 0.0001) and neutrophils (r =  − 0.49, p < 0.0001) (Fig. 9A); NRP1 had positive correlation with activated NK cells (r = 0.40, p < 0.0001), M1 macrophages (r = 0.37, p = 0.0206), resting dendritic cells (r = 0.34, p = 0.0264), and negative correlation with resting NK cells (r =  − 0.38, p = 0.0140) and neutrophils (r =  − 0.62, p < 0.0001) (Fig. 9B); COL4A1 had positive correlation with activated NK cells (r = 0.42, p < 0.0001), M1 macrophages (r = 0.42, p < 0.0001), and resting dendritic cells (r = 0.32, p = 0.0395), and negative correlation with resting memory CD4 T cells (r =  − 0.36, p = 0.0204), resting NK cells (r =  − 0.41, p < 0.0001), and neutrophils (r =  − 0.65, p < 0.0001) (Fig. 9C). These results indicated certain types of immune cells are dysregulated in the setting of IgAN.

## Discussion

With the biomedical research entering into the big data era, steadily increased number of algorithms and analysis tools are proposed by different research groups each year; moreover, multiple types of omics data about a specific type of disease are also repeated assessed by different groups. To obtain full insights into the systemic and pathological effects of omics data, an integrative bioinformatics analysis of these datasets by using the effective algorithms or tools have become the top priority, which can facilitate the further exploration by “Wet” experiments.

In present study, we employed limma analysis, overlap genes omitting and WGCNA to obtain DEGs from 1 blood-derived dataset and a glomeruli-derived dataset, and a total of 195 DEGs were found. Then we found 3 upregulated and 3 downregulated DEGs via checking of expression pattern and clinical features correlation. Finally, through the external dataset and an IgAN cohort validation, 3 genes including ORMDL2, NRP1, and COL4A1 were confirmed with the ability of IgAN discrimination, and the highest AUC was found by COL4A1, which is 97.14%.

During the datasets screening process, we also found another 2 datasets (GSE35488 and GSE14795) related with IgAN in the GEO website. In GSE35488 [21], RNA from tubulointerstitial compartments was extracted and processed for microarray analysis and the authors used these tissues for screening for proteinuria related DEGs. Finally, they identified an albumin-regulated 11-gene signature, which is shared in all forms of glomerulonephritits, including IgAN and membranoproliferative glomerulonephritis. Due to the poor gene clustering (Additional file 10) and inaccurate tissue derivation, this dataset was omitted. For GSE14795 [22], the whole blood cells from 12 IgAN patients were used for analysis using a specific commercial system, and DEGs were identified using SAM method, however, the DEGs obtained by us using the same SAM method showed significant difference from those reported in the papers, and we guess that the data from this GEO dataset is incomplete.

In the DEGs discovery process, we found 5 genes are of clinical significance. Among them, ORMDL2 is considered as a negative regulator of sphingolipid synthesis and is predicted to be located in the endoplasmic reticulum. Clarke et al. [23] reported that ORMDLs are able to restrain sphingolipid metabolism, thereby limiting levels of dangerous metabolic intermediates that can interfere with essential physiological processes such as myelination, whereas Bugajev et al. [24] demonstrated that ORMDL2 deficiency could affect mast cell signaling during the systemic anaphylactic reaction. Due to an abnormal sphingolipid metabolism could contribute to various pathologic processes, including kidney diseases [25], and activated mast cells were revealed according to the immune infiltration results, it is highly possible that ORMDL2 may an important role in IgAN. NRP1, a transmembrane receptor protein, has been reported to play an important role in several kidney diseases, including regulation of apoptosis and cytoskeleton organization of cells located in glomerular during the process of diabetic nephropathy [26], and prediction of renal outcome in patients with lupus nephritis [27]. COL4A1 encodes the α1 chain of collagen IV, which a major component of basement membranes and its mutation could result in glomerulocystic kidney disease [28–30]. According to previous reports [31, 32], macrophage can directly contribute to collagen generation in wound healing and other pathological conditions. They suggested that primary function of type VI collagen secreted by macrophages could be cell–cell (immune cells) and cell–matrix interactions modulation and production of type VI collagen is a marker for a nondestructive, matrix-conserving macrophage phenotype that could profoundly influence physiological and pathophysiological conditions in vivo. PKP4 is considered to play a role as a regulator of Rho activity during cytokinesis and is confirmed as one of DEGs in glomerular diseases reported by Ding et al. [33]. Most recently, Raby found that PKP4 is presented in the urine exosome in patients with Autosomal Dominant Polycystic Kidney Disease (ADPKD) as poor treatment responder biomarker [34]. In our cohort validation, we found that PKP4 is also upregulated in IgAN compared to normal controls, and it is omitted due to inconsistent expression pattern between the datasets and our patient cohort. HSPA8 is a chaperone protein with important roles in various cellular processes, including autophagy [35] and protection from reactive oxygen species (ROS) production by mitochondria during inflammatory conditions [36]. Kim et al. [37] reported that HSPA8 could be used as an effective and sensitive non-invasive biomarker for the assessment of nephrotoxicity, whereas Wen et al. [38] suggested that possible the role of HSPA8 in organ fibrosis in diabetic kidney disease (DKD) patients. In a recent study by Lin et al. [39], downregulated HSPA8 was found in IgAN patients, however, we did not find an obvious difference on HSPA8 in our discovery datasets. Therefore, further confirmation using more subjects might be carried out. No report was found on the role of ST13 in the kidney disease, whereas most of studies about ST13 are about the cancer biology [40]. Furthermore, ST13 was found with no significant downregulation in our validation cohort. We attributed the reasons that limited patient number or possible the error in the microarray analysis.

For the immune infiltration results, we found that differentially ratio of naive B cells (p = 0.016), resting memory CD4 T cells (p < 0.001), resting and activated NK cells (p = 0.021 and < 0.001), M1 and M2 macrophages (p = 0.016 and 0.020), activated mast cells (p = 0.038), and neutrophils (p < 0.001) between control and IgAN. B cells may be involved in the production of galactose-deficient IgA1(Gd-IgA1) and its antibodies in IgA nephropathy [41], and decreased number of naive B cell could be resulted by more of activated B cells. Moreover, Eijgenraam et al. [42] showed previously that dendritic cells of IgAN patients have an impaired capacity to induce IgA production in naive B cells. As heterogeneous cells of the innate immune system, macrophages can fluidly modulate their phenotype in response to the local microenvironment, and macrophage polarization is found during chronic kidney disease (CKD) [43], therefore, it is reasonable to observe elevation of M1 and M2 macrophages in IgAN. Moreover, activated mast cells mediated antibody production [44], neutrophils caused glomerular injury [45], NK induced hematuria [46] and T cell-induced Gd-IgA1 synthesis elevation [47] are reported according to previous reports during the IgAN.

In addition, according to the results from ceRNA network, miR-135a-5p and miR-135b-5p are found significantly correlated with the gene ORMDL2 found here, in the recent studies by Pawluczyk et al. [48] and Min et al.[49], miR-135a-5p showed differentially expressed in IgAN patients, whereas miR-135b-5p was confirmed as the differentially expressed miRNA using the urinary exosome samples from IgAN patients. Moreover, circulating miR-29a [50] and urinary exosome derived miR-29c [49] are also found differentially expressed in IgAN according to previous studies.

There are also some limitations in present study. First, the number of validation cohort is limited and no follow-up information is available, thereby resulting in possible the bias on the results and inability of examination of the genes in the prognosis. Second, although several immune cells were found dysregulated and the results were consistent with previous reports, the detailed function of these immune cells and the exact molecular events during the IgAN remains unknown. Therefore, further clinical validation with a large cohort of patient and experimental verification of these genes are required in near future.

## Conclusions

In summary, we demonstrated here that significantly upregulated DEGs: ORMDL2, NRP1 and COL4A1 could be served as the diagnostic marker for IgAN and dysregulated immune cell infiltration hinted possible the immune system intervention points in the setting of IgAN.

## Supplementary Information

**Additional file 1**: Supp1-Differentially expressed genes. Differentially expressed genes obtained after the screening process.

**Additional file 2**: Supp2-WGCNA color module genes. Detailed gene information form the specific WGCNA color module

**Additional file 3**: Supp3-Share up- and downregulated genes. Detailed information of the shared up- and downregulated genes.

**Additional file 4**: Supp4-eGFR and proteinuria correlated up genes. Detailed information of the eGFR and proteinuria correlated upregulated genes.

**Additional file 5**: Supp5-eGFR and proteinuria correlated down genes. Detailed information of the eGFR and proteinuria correlated downregulated genes

**Additional file 6**: Supp6-ceRNA network. Detailed mRNA, miRNA and cirRNA gene information of the ceRNA network.

**Additional file 7**: Supp7-CMAP results. Detailed information of target genes and related drugs.

**Additional file 8**: Supp8-CIBERSORT-Results. Detailed information of p-value of the CIBERSORT results.

**Additional file 9**: Supp9-correlation map results. Detailed information of the correlation parameters from CIBERSORT results.

**Additional file 10:** **Fig. S1**. Quality control report on the dataset screening process; For the datasets obtained from GEO that met the search criteria, we performed sample normalization (black for the control group and rose red for the IgAN group in box plots), clustering (dendrograms) and PCA analysis to obtain better quality samples. As shown in the following Figure, GSE73953 (A), GSE93798 (B) and GSE37460 (C) datasets were relatively homogeneous according to the sample expression abundance with well distinguished 2 groups of samples and no abnormal values from PCA results. However, for the dataset GSE35488 (D) and GSE14795 (E), bad clustering results from the dendrogram results and no discrimination could be found from PCA results. Furthermore, no effective values are found in GSE14795 according to the Venn map and using “limma” package. Therefore, GSE35488 and GSE14795 datasets were not included in the analysis.

**Additional file 11: Table S1**. Detailed information about the platform and sample information of the included microarray datasets (GSE73953, GSE93798 and GSE37460).

## Data Availability

The datasets supporting the conclusions of this article are included within the article and its additional files.
