# Supplementary figures and images for: Identification of blood-based key biomarker and immune infiltration in Immunoglobulin A nephropathy by comprehensive bioinformatics analysis and a cohort validation
Source: J Transl Med. 2022 Mar 29;20:145. doi: 10.1186/s12967-022-03330-w (PMC8966267; doi:10.1186/s12967-022-03330-w)

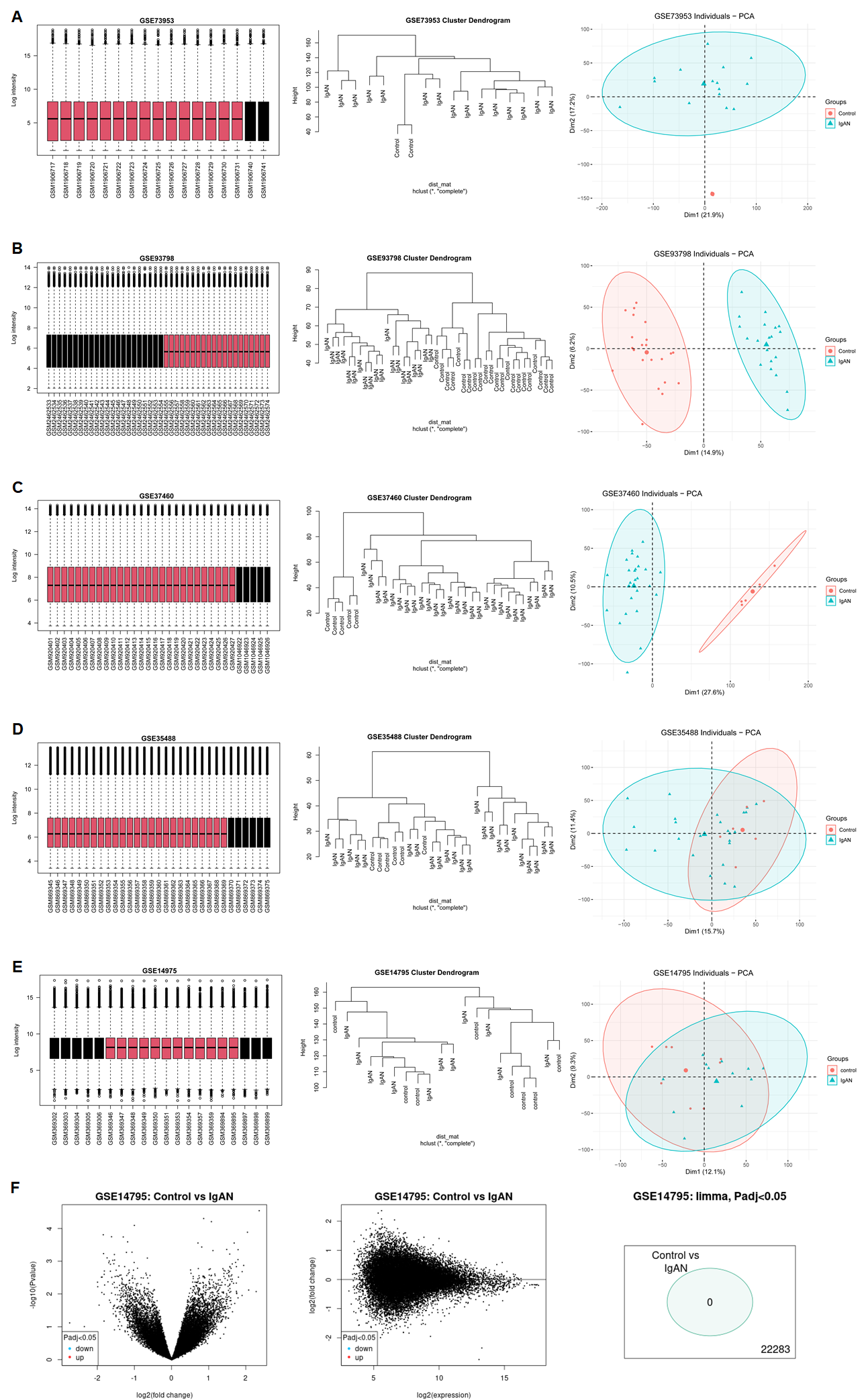

Supplement: Supplementary file 10 — Additional file 10: Fig. S1. Quality control report on the dataset screening process; For the datasets obtained from GEO that met the search criteria, we performed sample normalization (black for the control group and rose red for the IgAN group in box plots), clustering (dendrograms) and PCA analysis to obtain better quality samples. As shown in the following Figure, GSE73953 (A), GSE93798 (B) and GSE37460 (C) datasets were relatively homogeneous according to the sample expression abundance with well distinguished 2 groups of samples and no abnormal values from PCA results. However, for the dataset GSE35488 (D) and GSE14795 (E), bad clustering results from the dendrogram results and no discrimination could be found from PCA results. Furthermore, no effective values are found in GSE14795 according to the Venn map and using “limma” package. Therefore, GSE35488 and GSE14795 datasets were not included in the analysis. [file 12967_2022_3330_MOESM10_ESM.tif]
