# Supplementary material for: Identification of blood-based key biomarker and immune infiltration in Immunoglobulin A nephropathy by comprehensive bioinformatics analysis and a cohort validation
Source: J Transl Med. 2022 Mar 29;20:145. doi: 10.1186/s12967-022-03330-w (PMC8966267; doi:10.1186/s12967-022-03330-w)
Supplement: Supplementary file 11 — Additional file 11: Table S1. Detailed information about the platform and sample information of the included microarray datasets (GSE73953, GSE93798 and GSE37460). [file 12967_2022_3330_MOESM11_ESM.docx]

**Supplementary table 1. Detailed information of the included datasets**

| Series | Platform | Platform Name | Sample information |
| --- | --- | --- | --- |
| GSE37460 | GPL11670，GPL14663 | Affymetrix Human Genome U133 Plus 2.0 Array, Affymetrix GeneChip Human Genome HG-U133A Custom CDF | 25 healthy control and 29 IgAN glomeruli tissues. |
| GSE73953 | GPL4133 | Agilent-014850 Whole Human Genome Microarray 4x44K G4112F | peripheral blood mononuclear cells (PBMCs) from 15 IgAN patients and 16 healthy controls |
| GSE93798 | GPL22945 | [HG-U133_Plus_2] Affymetrix Human Genome U133 Plus 2.0 Array | 22 healthy control and 20 IgAN glomeruli tissues. |
